# Automatic Detection of Galaxy Type From Datasets of Galaxies Image Based on Image Retrieval Approach

**DOI:** 10.1038/s41598-017-04605-9

**Published:** 2017-06-30

**Authors:** Mohamed Abd El Aziz, I. M. Selim, Shengwu Xiong

**Affiliations:** 1 School of Computer Science and Technology, Wuhan University of Technology, Wuhan, China; 2 National Research Institute of Astronomy and Geophysics, Astronomy Department, Cairo, Egypt; 3 Department of Mathematics, Faculty of Science, Zagazig University, Zagazig, Egypt; 4 Higher Institute of Technology, Department of Computer Science, Tenth of Ramadan City, Egypt; 5 Scientific Research Group in Egypt (SRGE), Cairo, Egypt

## Abstract

This paper presents a new approach for the automatic detection of galaxy morphology from datasets based on an image-retrieval approach. Currently, there are several classification methods proposed to detect galaxy types within an image. However, in some situations, the aim is not only to determine the type of galaxy within the queried image, but also to determine the most similar images for query image. Therefore, this paper proposes an image-retrieval method to detect the type of galaxies within an image and return with the most similar image. The proposed method consists of two stages, in the first stage, a set of features is extracted based on shape, color and texture descriptors, then a binary sine cosine algorithm selects the most relevant features. In the second stage, the similarity between the features of the queried galaxy image and the features of other galaxy images is computed. Our experiments were performed using the EFIGI catalogue, which contains about 5000 galaxies images with different types (edge-on spiral, spiral, elliptical and irregular). We demonstrate that our proposed approach has better performance compared with the particle swarm optimization (PSO) and genetic algorithm (GA) methods.

## Introduction

Astronomy has become an immensely data-rich field. For example, the Sloan Digital Sky Survey (SDSS) will produce more than 50,000,000 images of galaxies in the near future^1^. In turn, galaxy morphology can be used to provide an independent test of the two proposed scenarios for galaxy formation. Elliptical galaxies, for example, are believed to be formed through major mergers^2^, whereas disk-dominated galaxies cannot have undergone recent major mergers, as such mergers would have severely disrupted their shape^3^. Thus, the class of quenching models is sufficient to explain the full range of morphological types observed for quenched galaxies. For example, bars can be found in all types of disk galaxies, from the earliest to the latest stages of the Hubble sequence. Barred galaxies constitute a major fraction of all disk galaxies. A small number of galaxies that appear unbarred at visual wavelengths have actually been found to be barred when observed in the near infra-red. The three clearest cases are NGC 1566^4^, NGC 1068^5, 6^ and NGC 309^7^. De Zeeuw and Franx^8^ surveyed the literature for the dynamics of these objects. We are still far from a complete understanding of the dynamical structure of galaxies. Here, we will be able to do no more than scratch the surface of the majority of these problems.

The development of galaxy morphological schemes can be used to successfully determine galaxy morphology via classification or image-retrieval methods. For example, the Deep Neural Network (DNN) algorithm has been used to classify the Galaxy Zoo (e.g. ref. 9). This method minimizes the sensitivity to changes in the scaling, rotation, translation and sampling of an image by using a rotation-invariant convolution. The results of this method is better than 99% with respect to human classification; however, as human classification has several associated errors, in turn the DNN approach also suffers from the same errors^10^. In ref. 11, the random forest method was used to classify an *HST*/WFC3 image containing 1639 galaxies, which identified disturbed morphologies using multimode, intensity and deviation statistics. Additionally^12^, proposed a method that consists of two stages: first, feature extraction (shape, color and concentration) of galaxy images from the SDSS DR7 spectroscopic sample, followed by the classification of these features using a support vector machine.

The authors in ref. 10, proposed a different approach called MORFOMETRYKA, which used the Linear Discriminant Analysis (LDA) algorithm to classify various features (concentration, asymmetry, smoothness, entropy and spirality) extracted from the galaxy images. The results of their approach were better than 90% based on 10-fold cross validation to classify a galaxy as either an elliptical or a spiral.

These galaxy classification methods have provided powerful results. However, there is another trend to deal with galaxy images, i.e. to determine the most similar images to query image, not classify them into groups only, therefore, the image retrieval techniques are needed^13^.

The image-retrieval method is a computer system for browsing, searching, detecting and retrieving images from a large database of digital images^14^. The content-based image retrieval (CBIR) approach is one of the most commonly used image retrieval methods^15^, which aims to avoid the use of textual descriptions and instead retrieves images based on similarities in their content. Relevant content can be information related to image patterns, colors, textures, shape and location^16^.

Such image content is obtained by using feature-extraction methods, which is then saved in a database. To answer a queried image, the similarity between stored features and the features of a queried image (extracted using the same method) is computed and used to determine the closest between the images. However, the CBIR approach is a challenging problem for galaxy images, because there is a large number of galaxy images and determining the most relevant images from a large database becomes a non-trivial task.

Several methods have been applied to improve the quality of CBIR for galaxy images. Ref. 17 introduced a CBIR method for astronomical images which used a multi-resolution approach to compress the original images in sketches. These sketches (features) were compared with the features of the queried image through the use of correlation and symmetry functions^18^. Next, ref. 19 proposed a CBIR method which summarized and indexed the Zurich archive of solar radio spectrograms. The summarized step was performed by clustering the content of an image into groups (regions) by using the same texture feature, which were represented by a set of parameters (location, a texture roughness and region extensions). The indexing step was then performed by quantizing these regions.

In general, the previous methods consider either the shape, the texture features or the color, or both of them (color/texture, color/shape and shape/texture), but not all of them. Moreover, not all of the extracted features are important: some may be redundant/irrelevant, which in turn reduce the quality of the classification or image-retrieval results. To address this, the aim of this paper is to introduce a new machine-learning approach for the retrieval of galaxy images. Our approach avoids the limitations of previous methods by extracting the shape, color and texture features from galaxy images, and then determining the most relevant features and ignoring other features by using the *K*-NN classifier as measure of the quality of the features which selected by Sine Cosine algorithm (SCA).

The proposed approach consists of two stages: training and image retrieval. In the training stage there are two steps: the first is feature extraction, where the color, shape and texture features are extracted from a dataset of galaxy images. The second step is feature selection, which is performed based on the modified sine cosine algorithm^20^ that selects the most relevant features using the classification accuracy as a fitness function. In the second stage, similar images to the queried image are returned by using the Euclidean distance as a measure.

## Feature extraction

In this section, visual features such as color, texture and shape are introduced^15^.

### Color Feature Extraction

The color of an image is one of the most widely used features in image retrieval and several other image-processing applications. It is a very important feature since it is invariant with respect to scaling, translation and rotation^21^. Therefore, the aim of any color feature extraction method is to represent the main colors of the image content (red, green, and blue, i.e. RGB) and then use these color features to describe the image and distinguish it from other images. RGB colors used in this study were obtained by converting from the SDSS color system using the Maxim DL astronomical software^22^.

The color histogram is one of the most well-known color features used for image feature extraction^23, 34^, which denotes the joint probability of the intensity of an image. From probability theory, a probability distribution can be uniquely characterized by its moments. Thus, if we interpret the color distribution of an image as a probability distribution, moments can be used to characterize the color distribution. The moments of the color distribution are the features extracted from the images; if we denote the value of the *i*th color channel at the *j*th image pixel as *P*_*ij*_, then the color moments can be defined as refs 23 and 24:

- The first-order moment (the mean):
  1 $${E}_{i}=\frac{1}{N}\sum _{j=1}^{N}\,{P}_{ij}$$
- The second-order moment (the standard deviation):
  2 $${\sigma }_{i}=\sqrt{\frac{1}{(N-\mathrm{1)}}\sum _{j=1}^{N}\,{({P}_{ij}-{E}_{i})}^{2}}$$
- The third-order moment (skewedness):

3 $${s}_{i}=\sqrt[3]{\frac{1}{N}\sum _{j=1}^{N}\,{({P}_{ij}-{E}_{i})}^{3}}$$

### Texture Feature Extraction

The texture descriptor is an important feature that provides properties such as smoothness, coarseness and regularity^25^. Textures can be rough or smooth, vertical or horizontal. Generally, they capture patterns in the image data, such as repetitiveness and granularity.

There are several texture extraction methods, such as the discrete cosine transform (DCT), the discrete Fourier transform (DFT), discrete wavelet transform (DWT) and the Gabor filter^26, 27^. The Gray Level Co-Occurrence Matrix (GLCM) and Color Co-Occurrence Matrix (CCM) are the most commonly used statistical approaches used to extract the texture of an image^28^. These features include the contrast, correlation, entropy, energy and homogeneity, which are defined as:

- The contrast represents the amount of local variation in an image. This concept refers to pixel variance, and it is defined as:
  4 $$CN=\frac{1}{{(G-\mathrm{1)}}^{2}}\sum _{u=0}^{G-1}\,\sum _{v=0}^{G-1}\,{|u-v|}^{2}p(u,v)$$
- The correlation represents the relation between pixels in an image, which determines the linear dependency between two pixels and is defined as:
  5 $$CR=\frac{1}{2}\sum _{u=0}^{G-1}\,\sum _{v=0}^{G-1}\,\frac{(u-{\mu }_{u})(v-{\mu }_{v})}{{\sigma }_{u}^{2}{\sigma }_{v}^{2}}p(u,v)+1$$
- The energy (*En*) represents the textural uniformity, where large values of *En* indicate a completely homogeneous image.
  6 $$En=\sum _{u=0}^{G-1}\,\sum _{v=0}^{G-1}\,p{(u,v)}^{2}$$
- The entropy (*ET*) measures the randomness of the intensity distribution. It is inversely correlated to *En*, and is defined as:
  7 $$ET=\frac{1}{2log(G)}\sum _{u=0}^{G-1}\,\sum _{v=0}^{G-1}\,p(u,v)\,lo{g}_{2}\,p(u,v)$$
- The homogeneity (*H*) is used to measure the closeness of the distribution, where large values *H* indicate that the image contrast is low. The definition of *H* is given in the following equation:

8 $$H=\sum _{u=0}^{G-1}\,\sum _{v=0}^{G-1}\,\frac{p(u,v)}{1+|u-v|}$$

where *u*, *v* are the coordinates of the co-occurrence matrix, *G* is the number of grey levels, and *μ*_*u*_, *μ*_*v*_, *σ*_*u*_, and *σ*_*v*_ are the mean values and the standard deviations of the *u*th row of the *v*th column of the co-occurrence matrix, respectively.

### Shape Feature Extraction

Shape features were extracted by using the contour moments defined mathematically as follows. Let *z*(*i*) be an ordered sequence that represents the Euclidean distance between the centroid and all *N* boundary pixels of the object. The *r*th contour sequence moment *m*_*r*_^14^ is defined as:

9 $${m}_{r}=\frac{1}{N}\times \sum _{i=1}^{N}\,{[z(i)]}^{r}$$

## Sine Cosine Algorithm

In this section, the sine cosine algorithm (SCA) is illustrated^20^, this algorithms is a new meta-heuristic algorithm which used either the sine or cosine function to search about the best solution. Consider the current solution *X*_*i*_, $(i=1,2,\ldots ,po{p}_{size})$ from the population of solutions is updated as in the following equation^20^:

10 $${X}_{i}={X}_{i}+{r}_{1}\times \,\sin ({r}_{2})\times |{r}_{3}P-{X}_{i}|$$

11 $${X}_{i}={X}_{i}+{r}_{1}\times \,\cos ({r}_{2})\times |{r}_{3}P-{X}_{i}|$$

The previous two equations were combined to update the solution that can be simultaneously by switching between the sine or cosine function^20^:

12 $${X}_{i}=\{\begin{array}{ll}{X}_{i}+{r}_{1}\times \,\sin ({r}_{2})\times |{r}_{3}P-{X}_{i}| & if\,{r}_{4} < 0.5\\ {X}_{i}+{r}_{1}\times \,\cos ({r}_{2})\times |{r}_{3}P-{X}_{i}| & if\,{r}_{4}\ge 0.5\end{array}$$

where *r*_1_, *r*_2_, *r*_3_ and *r*_4_ are random variables, *P* is the best solution, and |·| represents the absolute value^20^.

**Figure 1:**
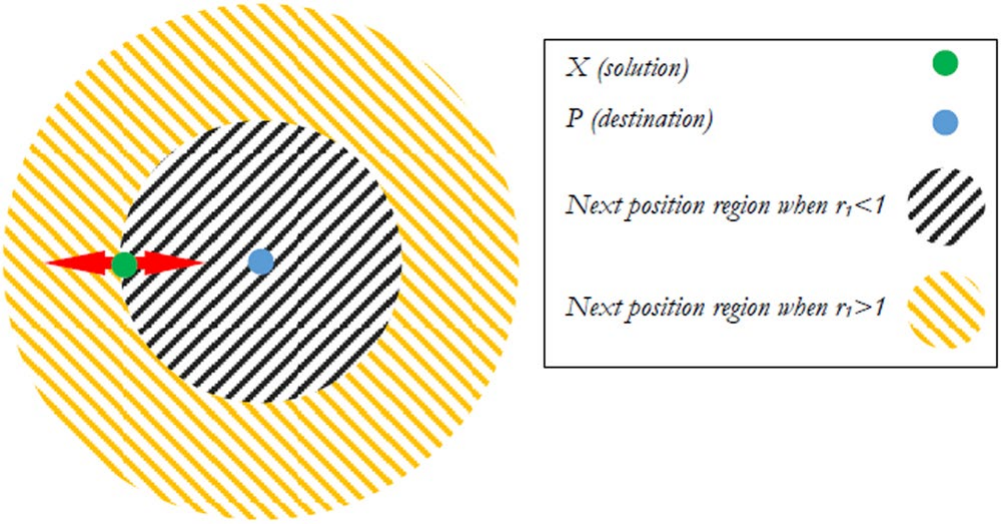
The Sine and Cosine functions effects on the next solution^20^.

Following ref. 20, each parameter was used to perform a specific task. For example, the *r*_2_ parameter defines the direction of *X*_*i*_ (i.e., towards or away from *P*), while *r*_3_ gives random weights to *P* in order to stochastically emphasize (*r*_3_ > 1) or deemphasize (*r*_3_ < 1) its influence when defining the distance. Next, *r*_4_ is responsible for switching between the sine and cosine functions in equation (12) ^20^. Finally, *r*_1_ was used to determine the next position regions (or movement direction), which could be either in the space between *X*_*i*_ and *P* or outside of this space, and it is also responsible for balancing between the exploration and exploitation to improve the convergence performance by updating its value as ref. 20:

13 $${r}_{1}=a-t\frac{a}{{t}_{max}}$$

where *t* is the current iteration, *t*_*max*_ is the maximum number of iterations, and *a* is a constant. Figure 1 shows how equation (12) defines a region between two solutions in the searched space.

## The Proposed Image Retrieval Approach

In this section, we investigate a new approach to galaxy image retrieval as illustrated in Algorithm 1. Our proposed approach consists of two stages: a training stage and the galaxy image retrieval stage.

In the first stage, the input is the dataset of galaxy images. Then the shape, texture and color features are extracted for each galaxy image *I*, which are combined into a feature vector *FV*_*I*_, where *I* is the current image. The next step in the training stage is to reduce the size of *FV* through using the Binary SCA (BSCA) algorithm (see Algorithm 2) to select the most relevant features. This process is performed by maximizing the accuracy of the *K*-NN classifier, which is used as a fitness function.

The BSCA starts by generating a random population of size *pop*_*size*_, and the output is the best solution *P* that points to the selected features (*Sel*_*Feat*_). The solution in the population of the BSCA algorithm is represented as a binary vector by using the sigmoid function which transforms a real number into a binary number as:

14 $${X}_{i}=\{\begin{array}{ll}1 & if\,S({X}_{i}) > \sigma \\ 0 & otherwise\end{array},\,S({x}_{i})=\frac{1}{1+{e}^{-{X}_{i}}}$$

where $\sigma \in [0,1]$ and *X*_*i*_ is the current solution (for example, the solution *X*_*i*_ = 001100 with six features means that the third and fourth features are selected).

After the solutions are converted to binary vectors, the fitness function is computed for each solution. The fitness function is defined according to the classification accuracy rate as:

15 $${F}_{i}=\frac{{N}_{C}}{{N}_{I}}\times 100$$

where *N*_*C*_ is the number of correctly predicted samples, and *N*_*I*_ represents the total number of images. The dataset is divided by using a 10-fold cross validation (CV), and then the *K*-NN algorithm predicts, using the label of the testing set, where the output from 10-fold CV is the average of accuracy through 10 runs.

The solution *X*_*i*_ is updated using equations (10) or (11) based on the value of *r*_4_. This process is repeated until the maximum number of iterations is reached, or there is only a small difference between ${F}_{i}^{old}$ and *F*_*i*_. The output of this stage is the global best solution *P*, which represents the optimally selected features *Sel*_*Feat*_.

The second stage starts by extracting the features of a queried image *FQ*, and then the same features corresponding to *Sel*_*Feat*_ are selected. Then the Euclidian distance is used to compute the similarity between *FQ* and *FV*

**Table:** 

**Algorithm 1** The Proposed approach For Galaxy Image Retrieval		
1:	Input: database of images, queried image.	
2:	Output: precision and recall.	
3:	Training stage:	
	•	Compute the feature vectors *FV*_*I*_ for all images in the database.
	•	Select features *Sel*_*Feat*_ = BSCA(*FV*).
	•	Update the set of features *FV* = *FV*(*Sel*_*Feat*_).
4:	Image retrieval stage:	
	•	Compute the feature vector *FQ* of the queried image *I*_*Q*_.
	•	Update *FQ* = *FQ*(*Sel*_*Feat*_).
	•	For {all **I**_*i*_ % in parallel techniques}
	•	Compute the distance between *FQ* and *FV*_*i*_ using the Euclidean distance *E* *Dist*_*i*_.
	•	end for
5:	Select the smallest distance from *E* *Dist* and determine the index *S*_*index*_ that satisfies *E* *Dist* < $\epsilon$.	
6:	Select from the database any images with index *S*_*index*_.	
7:	Compute the precision and recall.

**Table:** 

**Algorithm 2** Binary Sine Cosine Algorithm (BSCA)	
1:	Input: features of each image (*FV*).
2:	Initialize a set of solutions (*X*) with size *pop*_*size*_, and set the maximum number of iterations *t*_*max*_.
3:	**for** i = 1: *pop*_*size*_ **do**
4:	Convert *X*_*i*_ to a binary vector using equation (14).
5:	Compute the fitness function *F*_*i*_ based on the selected features from *FV* and using 10-fold cross-validation.
6:	**if** *F*_*i*_ < *F*_*P*_ **then**
7:	*F*_*P*_ = *F*_*i*_.
8:	*P* = *X*_*i*_.
9:	**end if**
10:	**end for**
11:	**repeat**
12:	Update *r*_1_, *r*_2_, *r*_3_, and *r*_4_.
13:	Update the position using equation (12).
14:	**until** (*t* < *t*_*max*_)
15:	Return the best solution *P* obtained so far as the global optimum *F*_*P*_.

, and the closest images to the query image are returned (based on the small difference or the required number of images).

## Experimental Results

We tested our proposed approach using the EFIGI catalogue, which consists of 4458 galaxy images^29^. We also compared the performance of our method with the particle swarm optimization (PSO)^30^ and genetic algorithm (GA)^31^ methods. The parameters used in each algorithm is given in Table 1. The common parameters between the three algorithms are the population size, the maximum number of iterations which was set to 20 and 100, respectively, and the maximum number of iterations used as the stopping criteria. The experiments were implemented in Matlab and run in the Windows environment with 64-bit support.

**Table 1:** The parameter settings of each algorithm.

Algorithm	Parameters	Value
BSCA	a	2
PSO	Inertia weight	0.5
	Maximum velocity	1.0
	Minimum velocity	−1.0
	Cognitive coefficient	1
	Cognitive coefficient	2
GA	cross probability of	0.7
	Mutation Percentage	0.4
	Mutation Rate	0.1

### Images Database

The EFIGI catalogue^29^ contains 16 morphological attributes that were measured by visual examination of the composite g, u, r color image of each galaxy, derived from the SDSS FITS images using^29^. The EFIGI catalogue merges data from standard surveys and catalogues (the Principal Galaxy Catalogue, SDSS, the Value-Added Galaxy Catalogue, HyperLeda, and the NASA Extragalactic Database). The bulge-to-disk ratio^32^ and the degree of azimuthal variation of the surface brightness were often used as discriminant parameters along the Hubble sequence. This is not surprising since the EFIGI classification scheme is very close to the RC3 system. The final EFIGI database is a large sub-sample of the local universe which densely samples. The EFIGI morphological sequence is based on the RC3 revised Hubble sequence (RHS), which we call the EFIGI morphological sequence (EMS).

Finally, all colors of the original data were used to create composite, “true color”, RGB images in PNG format with the Maxim DL astronomical software^22^, using the same intensity mapping for all RGB images.

### Performance measures

Two measurements were used to evaluate the performance of the proposed algorithm: the precision rate and the recall rate.

- The precision rate is defined as the ratio of the number of retrieved images similar to the queried image relative to the total number of retrieved images^28^.
  16 $$precision=\frac{p}{p+r}\times 100$$
- The recall rate is defined as the percentage of retrieved images similar to the query image among the total number of images similar to the queried image in the database^28^.

17 $$recall=\frac{p}{p+q}\times 100$$

where *p*, *q* and *r* are the number of relevant images retrieved, relevant images in the dataset which are not retrieved, and non-relevant images in the dataset which are retrieved, respectively.

## Results and Discussion

In order to assess the effectiveness of our approach, we used the leave-one-out cross-validation method, where each image in the dataset was considered as the queried image, and the process was repeated 4458 times. Also, we used the 1-NN method based on 10-fold cross-validation (CV), which was used to evaluate the subset of selected features. This classifier is a parameter-free feature and is easy to implement^33^. As discussed previously, the 10-fold CV works by dividing the dataset into ten groups, and the experiment was performed ten times by selecting one group as the test set and the remaining groups were used as a training set during each run. The output is the average of accuracy of the ten runs.

In general, we used color, texture and shape feature vectors for galaxy image retrieval. The total number of extracted features was 30, where nine features were extracted from the three colors RGB (three moments for each color), 20 texture features (four rotations for each measure) and one shape feature. The extracted feature vectors were applied to the feature selection method (in this study, we compared the BSCA, PSO and GA methods) to determine the relevant features.

The best selected features with their accuracy (the value of fitness function) are given in Table 2. From this table it can be seen that, the BSCA algorithm selects a small number of features with high accuracy followed by the PSO, however, the GA selects a large number of features with low accuracy. In addition, we observed that the more relevant features thatcontain more information and are used to distinguish between the classes are the third color moment, energy, homogeneity, entropy and contour. These features are common between the three algorithms, and all of them are selected by the proposed method.

**Table 2:** The selected features and their accuracy.

	No. of Features	Name of Selected Features	Accuracy
BSCA	12	Third Color moment (3), Energy(2), Homogenity(3), Entropy(3), Contour moment (1)	94.23
PSO	19	Third Color moment (3), Second Color moment (3), Contrast(2), Energy(3), Homogenity(2), Entropy(2), Contour moment(1)	93.59
GA	20	Third Color moment (3),Second Color moment (3), Contrast(4), Energy(4), Homogenity(4), Entropy(1), Contour moment(1)	92.95

**Figure 2:**
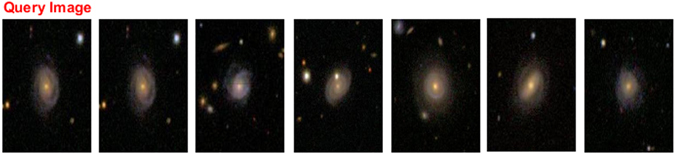
Galaxy image retrieval for a spiral galaxy^29^.

**Figure 3:**
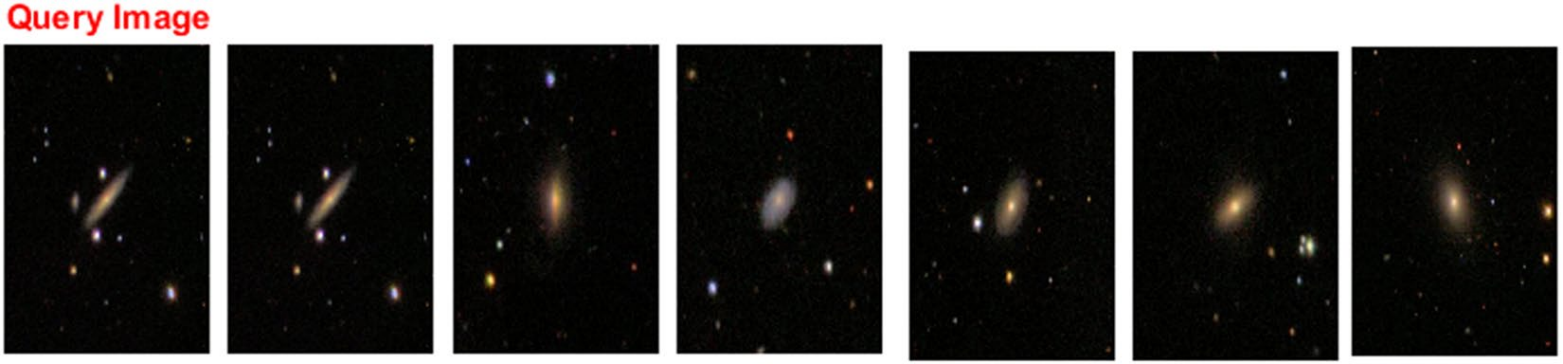
Galaxy image retrieval for an edge-on spiral galaxy^29^.

**Figure 4:**
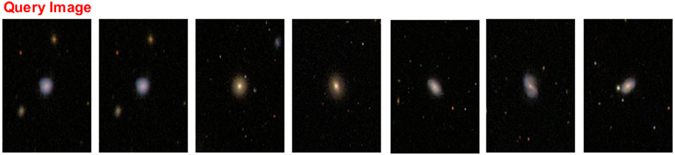
Galaxy image retrieval for an irregular galaxy^29^.

**Figure 5:**
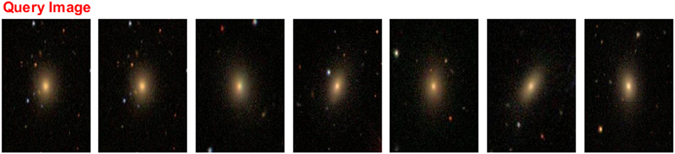
Galaxy image retrieval for an elliptical galaxy^29^.

The comparison results of our proposed method with other methods are illustrated in Figs 2, 3, 4 and 5 and Table 3. From Table 3, we can conclude that the proposed approach is better than PSO and GA in terms of precision and recall measures. The best results were obtained when the spiral-edge type was used as the queried image because they present the most regular structure, while the less accuracy occurs when the spiral type galaxy was tested.

**Table 3:** A comparison between the proposed approach and the PSO and GA methods for galaxy image retrieval.

Dataset	Proposed approach		PSO		GA	
	Recall	Precision	Recall	Precision	Recall	Precision
Elliptical	92.68	97.43	90	85.36	82.60	97.44
Spiral Edge	97.50	100	100	100	97.50	100
Spiral	96.87	79.48	91.42	96.96	96.66	74.35
Irregular	90.69	100	92.85	90.69	97.50	100
Avg. Time (s)	292.0		508.1		495.0	

Moreover, from Table 3, it can be seen that the proposed method is faster than the other two algorithms, which takes ~292.0 s (nearly half the time of the other algorithms) to select the best features. We note that the GA method takes less time to complete than the PSO algorithm. In general, the computing time is divided into three parts: the first is the time needed to extract features from the images (~375 s, where each image takes ~0.084). The second part is the time needed to select the most relevant features as in Table 3. The last part is the time needed to compute the matching, which requires ~0.0157 s in addition to the time need to extract the features of the queried image (~0.084).

Figures 2, 3, 4 and 5, show an example of the retrieval images for four galaxy types. In these figures, the five database images that are the closest to the queried image are given as the retrieval results.

In order to investigate the influence of the size of the training set when selecting the best features, the dataset was randomly divided into training and testing sets. The proposed method was then evaluated at three different sizes, i.e. 50%, 70% and 85% of dataset (the remaining is the test set). Our results are shown in Table 4, where it can be seen that the worst accuracy was obtained when the sizes of the training and test sets were equal. The best accuracy was achieved when the training set was 85% of the entire database (as expected: by increasing the size of training set, the accuracy also increases).

**Table 4:** The effect of the size of training set on the performance of the proposed approach for galaxy image retrieval.

Dataset	50/50		70/30		85/10	
	Recall	Precision	Recall	Precision	Recall	Precision
Elliptical	72.67	77.50	86.67	86.33	91.87	94.58
Spiral Edge	80.33	79.65	89.15	88.77	95.93	98.95
Spiral	83.72	68.60	87.60	70.96	93.37	75.28
Irregular	60.61	60.00	82.07	75.76	85.17	94.68
NO. Features/Accuracy	20/81.85		18/88.30		15/92.02	

Finally, from the previous results, we can conclude on two things: first is that the proposed approach for galaxy image retrieval is better than the PSO and GA algorithms in terms of recall, precision, accuracy and the time complexity. The second is that the most suitable method used to split the dataset (when selecting the best-fitting features) is the 10-fold CV, however, if the dataset is divided randomly then the most suitable size for the training set is in the range 85% to 90%.

## Conclusions

In this study, we proposed a machine learning approach for galaxy image retrieval used for the automatic detection of galaxy morphological types from datasets of galaxies images. The automated detection of galaxies types is very important to understand the physical properties of the past, present, and future of the universe, while also offering a means for identifying and analyzing peculiar galaxies that cannot be associated with a defined morphological stage on the Hubble sequence.

Our analysis was performed such that our approach automatically detected specific morphology types from different morphological classes without human guidance. The proposed algorithm was compared with the PSO and GA algorithms, and its performance was evaluated based on recall and precision. The results indicate the superior performance of our proposed approach.

Based on the promising results of the algorithm, our future work will attempt to further investigate its application to other complex problems in astronomy by modifying the proposed method.
